# The potential for Treg-enhancing therapies in transplantation

**DOI:** 10.1093/cei/uxac118

**Published:** 2022-12-23

**Authors:** Romy Steiner, Nina Pilat

**Affiliations:** Department of General Surgery, Medical University of Vienna, Vienna, Austria; Department of Cardiac Surgery, Medical University of Vienna, Vienna, Austria; Center for Biomedical Research, Medical University of Vienna, Vienna, Austria; Department of General Surgery, Medical University of Vienna, Vienna, Austria; Department of Cardiac Surgery, Medical University of Vienna, Vienna, Austria; Center for Biomedical Research, Medical University of Vienna, Vienna, Austria

**Keywords:** Treg, transplantation, tolerance

## Abstract

Since the discovery of regulatory T cells (Tregs) as crucial regulators of immune tolerance against self-antigens, these cells have become a promising tool for the induction of donor-specific tolerance in transplantation medicine. The therapeutic potential of increasing *in vivo*Treg numbers for a favorable Treg to Teff cell ratio has already been demonstrated in several sophisticated pre-clinical models and clinical pilot trials. In addition to improving cell quantity, enhancing Treg function utilizing engineering techniques led to encouraging results in models of autoimmunity and transplantation. Here we aim to discuss the most promising approaches for Treg-enhancing therapies, starting with adoptive transfer approaches and *ex vivo*expansion cultures (polyclonal vs. antigen specific), followed by selective *in vivo*stimulation methods. Furthermore, we address next generation concepts for Treg function enhancement (CARs, TRUCKs, BARs) as well as the advantages and caveats inherit to each approach. Finally, this review will discuss the clinical experience with Treg therapy in ongoing and already published clinical trials; however, data on long-term results and efficacy are still very limited and many questions that might complicate clinical translation remain open. Here, we discuss the hurdles for clinical translation and elaborate on current Treg-based therapeutic options as well as their potencies for improving long-term graft survival in transplantation.

## Introduction

Recent innovations in surgical techniques and therapeutics led to remarkable improvements considering short-term graft survival in solid organ transplantation (SOT) patients. Long-term graft survival, however, has not improved much in the last decades [1]. Despite continuous immunosuppression patients experience late graft loss due to chronic rejection that cannot be properly controlled. Moreover, they suffer from increased morbidity and mortality associated with the unspecific suppression of the immune system and the toxicity of immunosuppressive drugs [2, 3]. Therefore, the induction of immunological tolerance, namely the acceptance of an allograft in the absence of chronic immunosuppression, is the ultimate goal in transplantation. In rare cases of liver and kidney transplantation a state of ‘operational tolerance’ with stable graft function without chronic immunosuppression was achieved; however, occurrence was unpredictable, spontaneous, and late after transplantation [4–6].

**Figure 1:**
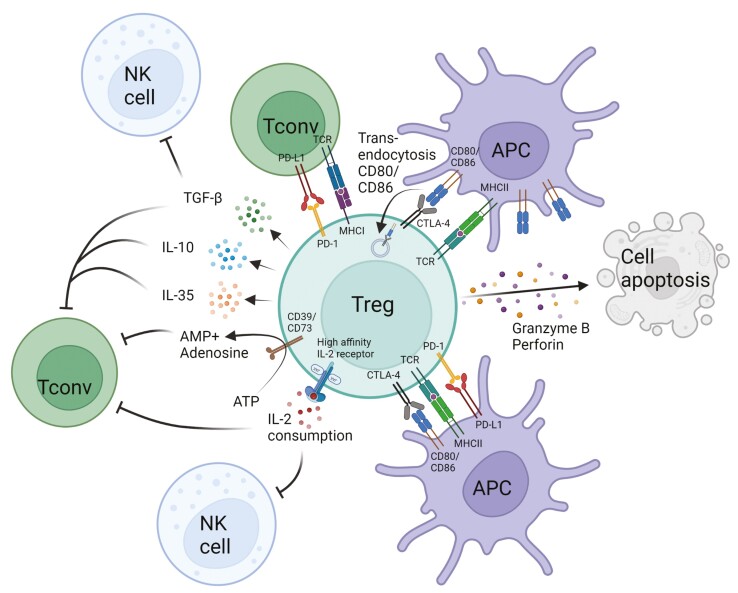
approaches of Treg-based therapy in transplantation medicine. (APC, antigen-presenting cells; PBMCs, peripheral blood mononuclear cells; created with BioRender.com)

Regulatory immune cells have been recognized as key players in immune homeostasis and have been suggested as perfect candidates for deliberate induction of donor-specific tolerance in SOT. As a result, several approaches favoring tolerance induction through regulatory T-cell (Treg) enhancement have been described in preclinical models and clinical trials (Fig. 1).

### Heterogeneity of regulatory T cells

The first evidence of so-called suppressor T lymphocytes goes back to 1969 [7]; however, their existence was under debate for the next 25 years. Since the ‘official’ discovery of Tregs in 1995 as a small CD4 + T-cell subpopulation with high levels of IL-2Rα (CD25) expression and the capacity of protecting thymectomized mice from autoimmunity [8], we learned a lot about the crucial role of Tregs in immune responses [9, 10]. A major break-through came in 2003 when the X-linked gene *FOXP3* was identified as main transcription factor responsible for Treg phenotype and function. Disruption of *FOXP3* leads to early onset of multi-organ inflammation and fatal autoimmune disease in mice and men [11–13]. For example, mutations in *FOXP3* result in the development of immunodysregulation polyendocrinopaty enteropathy X-linked (IPEX) syndrome in male humans [14]. Utilizing a mouse model homologue of FOXP3 deficiency (scurfy mice), the first rescue experiments using adoptive Treg transfer were successfully performed, paving the way for these cells to be applied in therapy of IPEX patients and other autoimmune diseases with dysfunctional FOXP3 expression [13, 15, 16].

Tregs comprise about 5-10% of circulating CD4 + T cells and are characterized by the constitutive and high expression of CD25 and FOXP3 [17]. While FOXP3 expression in mice is specific for Tregs, in humans it has been shown that CD4 + CD25-effector T cells (Teff) are capable of transiently expressing the transcription factor [18]. Moreover, it has been demonstrated that FOXP3 expression in human Tregs, contrary to murine Tregs, is not homogenous with several splicing variants that have been identified in the last years. There are two main isoforms that are expressed at comparable levels by human Tregs and are distinguished by the full-length expression of FOXP3 or lack of exon 2. The deficiency of exon 2 leads to the inability of FOXP3 interaction and inhibition of RORα and RORγt contrasting the development of Th17 cells [19]. A third isoform lacking the exon 2 as well as exon 7 has been described as Th17 differentiation facilitating [20]. In addition, it has been shown that loss of the full length FOXP3 is associated with impaired lineage stability [20]. Inflammatory conditions, like in autoimmune diseases may result in conversion of FOXP3 + Tregs into Th17 cells, contributing to disease progression and impairing immune homeostasis, therefore stable full length expression of FOXP3 is a prerequisite for Treg cell therapy [21]. Analysis of human splicing variants during Treg isolation and later adoptive cell transfer is hindered not only by the fact that FOXP3 is an intracellular marker, but also by the limited options for anti-FOXP3 antibodies recognizing exon 2 or Δ2 a as well as no available antibody against exon 7 or Δ7 [22]. However, in humans an inverse correlation of FOXP3 expression and expression of the IL-7 receptor (CD127) α chain was identified, establishing CD4 + CD25 + CD127low as surface markers for human Treg identification [23, 24]. In 2009, Miyara et al. classified human Tregs based on their expression levels of FOXP3 and CD45RA in naïve/resting (FOXP3low CD45RA+), effector-type (FOXP3high CD45RA-) and cytokine-producing (FOXP3low CD45RA-) Tregs [25]. In fact, there are studies demonstrating superior proliferation capacity of human naïve CD45RA + Tregs with higher stability and suppressive function when compared to effector or memory phenotype Tregs [26]. However, although overall Treg numbers increase, the amount of naïve Tregs decreases with age, complicating the use of CD45RA + Tregs for adoptive cell transfer in adults and older patients [27].

In addition, circulating Tregs can be divided according to their origin of differentiation since they develop in the thymus (tTreg), the periphery (pTreg) as well as *in vitro* (iTregs) in presence TGF-β and IL-2 [9]. While thymus derived Tregs mainly recognize self-antigens, pTregs which develop from CD4 + conventional T cells (Tconv) have been shown to recognize ‘non-self’, showing a similar T-cell receptor (TCR) repertoire as conventional T cells. While in mice the differentiation between tTregs and pTregs is possible by neuropilin 1 (NRP1) expression, in human no marker has been identified yet [28]. Recent literature suggests the expression of Helios to discriminate between tTregs and pTregs in human, although its validity remains contentious [29–31]. Besides, different papers state that the analysis of epigenetic DNA methylation levels on the non-coding conserved region of the *FOXP3* gene, namely the Treg-specific demethylation region (TSDR), is still the only reliable way to distinguish between these subsets in humans [32, 33].

### Suppressive capacity of regulatory T cells

**Figure 2:**
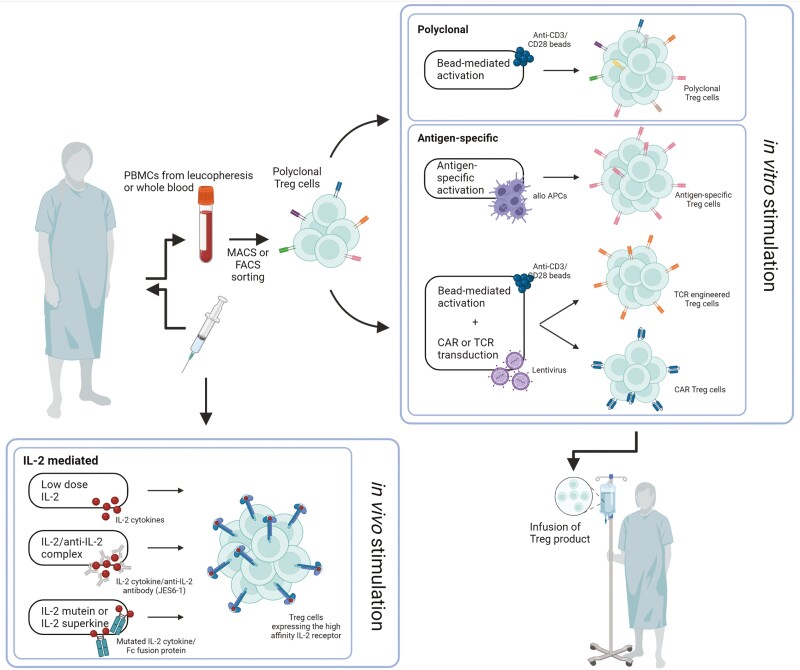
mechanisms of Treg-mediated suppression. (APC, antigen-presenting cell; Tconv, conventional T cell; NK cell, natural killer cell; created with BioRender.com)

Tregs suppress immune responses by cell-contact dependent and independent mechanisms (Figure 2). These processes include the secretion of anti-inflammatory cytokines such as TGF-β, IL-10, and IL-35 to suppress Tconv and natural killer (NK) cells [34–36]. In addition, Tregs are capable of excess IL-2 consumption by the expression of the IL-2 high-affinity receptor complex IL-2αβγ, limiting the availability of IL-2 for other IL-2 responsive subsets (NK, CD8, etc.) [8, 37, 38]. The expression of cell surface receptor like cytotoxic T lymphocyte antigen 4 (CTLA-4) on Tregs negatively regulates antigen-presenting cell (APC) function by binding to CD80/CD86 and subsequent blockade of CD28 ligation by Tconv [39, 40]. Tregs have been shown to deplete CD80/86 by trogocytosis [41, 42], therefore actively reducing costimulatory molecules on APCs. Furthermore, the release of lytic proteins such as perforin and granzyme B are able to directly kill target antigen expressing APCs [43]. The surface expression of CD39 and CD73 on Tregs mediates conversion of ATP to AMP causing further reduction of Tconv proliferation [44].

Notably, a variety of these Treg-mediated suppressive mechanisms occur in an antigen unspecific manner, resulting in suppression of Teff cells with diverse specificities [45]. Tregs are capable of shaping a microenvironment promoting the attraction of other immunosuppressive cell populations. This effect was first described by Gershon and Kondo in 1971 as ‘infectious tolerance’ and suggests that adoptively transferred Tregs may not need to persist indefinitely, but long enough to transfer the suppressive capacities to other immune cells [46]. In 1993 the Waldmann group studied this effect in a murine skin transplantation setting. Thymectomized mice (CBA/Ca) received a tolerizing protocol utilizing an induction therapy combining CD4 as well as CD8 non-depleting antibodies and a allogeneic skin graft (B10.BR). Upon challenge with naïve recipient-type lymphocytes, infectious tolerance was conferred to freshy infused cells and sustained tolerance was shown by indefinite survival of another donor-type skin graft. If CD4+, but not CD8 + T cells were depleted before naïve lymphocyte infusion, however, skin grafts were rejected, suggesting again a profound role of CD4 + T cells in tolerance induction. Furthermore, they showed that coexistence of tolerized host and freshly transferred naïve cells from the same mouse strain for 2 weeks *in vivo* led to tolerance against a second same-donor skin graft (B10.BR) even after selective depletion of tolerized host cells, impressively demonstrating the effects of ‘infectious tolerance’ in transplantation [47]. Notably, infectious tolerance was also seen in humans were Tregs conveyed suppressor activity to conventional CD4 + T helper cells [48], again proving the importance of Tregs for cell-based therapies.

## Regulatory T cells: therapeutic approaches

### Adoptive cell therapy

There are several open questions regarding the best *in vitro* expansion approach for adoptive cell therapy, regarding Treg source, isolation method, culture conditions and specificity. Tregs can be isolated from the donor, recipient, or even third-parties (‘off-the-shelf product’) and isolation sites include peripheral blood mononuclear cells (PBMCs), umbilical cord-blood (UCB), and (pediatric) thymi. Isolation techniques and markers used for sorting of the starting cell populations as well as conditions for expansion cultures also vary between published studies. Moreover, the right cell dose in clinical (or pre-clinical *in vivo*) studies has yet to be determined and cannot simply be extrapolated from *in vitro* suppressor assays.

#### Treg source and isolation

Whereas most clinical studies use PBMC-derived cells, it has been reported that comparable frequencies of Tregs can be found within the UCB. Moreover cells isolated from UCB are largely naïve, since the UBC is almost devoid of CD25 + antigen-experienced memory and effector T cells, making it a highly suitable source for Treg isolation in the clinical setting [49, 50]. One notable drawback of using UBC is the very low yield of Tregs per unit. A challenge that may be overcome by pooling multiple donors [51]. Pediatric thymi routinely obtained during heart surgery are in addition a valuable source for Tregs [52]. More recently, Lombardi et al. were able to generate a good manufacturing practice (GMP)-compatible protocol for the expansion of pediatric thymus-derived CD3 + CD4 + CD25 + CD127- (Tregs) as well as CD3 + CD4 + CD25 + CD127- CD45RA + (RA + Tregs) cells, paving the way for future clinical application [53]. Indeed, a phase I/II clinical trial for prevention of heart transplant rejection in children using autologous Tregs isolated from thymic tissue is currently ongoing (NCT04924491). Besides the UCB and the thymus, peripheral blood (PB) remains the main source used for Treg cellular products in clinical trials [54]. In theory, Tregs for adoptive cell therapy can be obtained from the recipient, the donor or even unrelated third-parties. In a pre-clinical study our group could directly show the superiority of recipient-based Tregs for tolerance induction via hematopoietic chimerism in transplantation [55]. Moreover, autologous (recipient-derived) Treg therapy has been demonstrated to be feasible and safe in numerous clinical studies [56, 57]. Interestingly, allogeneic (donor-derived) Tregs have shown superior effectiveness in preventing graft versus host disease (GvHD) after allogeneic stem cell transplantation [58]. Hoffman et al. developed the first GMP-compliant isolation method for magnetic sorting of CD4 + CD25 + T cells from standard leukapheresis products using the CliniMACS system (Miltenyi Biotec, Bisley, UK) [59]. This system utilizes a two-step magnetic bead isolation involving a CD8+/CD19 + depletion step followed by the enrichment of CD25 + cells via positive selection. However, multiple parameters for stricter Treg selection are not applicable in this system, representing an important limitation of this approach. In addition, the purity of bead-isolated Tregs regarding their FOXP3 expression is reportedly limited to about 80% raising the concern of contamination with activated Teff cells that potentially cause graft damage upon injection into the patient [60]. Therefore fluorescence activated cell sorting (FACS) of Tregs based on selected cell markers by flow cytometry gained importance as a method allowing high purity Treg (>99%) isolation [61]. In recent times effort was made in developing a GMP-compliant closed FACS-system for clinical application, allowing the use of multiple parameters and quality of expression during Treg isolation, e.g. CD25high, CD62Lhigh, CD127low, CD45RA, which is not possible using the European Medicines Agency (EMA) approved CliniMACS system. In the human setting it was demonstrated that only Treg cultures originated from CD4+, CD25high, CD45RA + sorted cells maintained FOXP3 stability as well as high suppressive capacity following *in vitro* cell culture [26, 62]. Based on these results, FACS sorting for Treg isolation is already part of clinical kidney transplantation trials (NCT02088931; NCT03867617) [63, 64].

#### *In vitro* expansion

Besides the challenge of manufacturing highly pure Treg cell products, the amount of cells needed for adoptive transfer is another hurdle to overcome for efficient Treg cell therapy. Based on mouse models for tolerance induction, it is suggested that *in vivo* Treg numbers need to be increased by 33% in order to reach a Treg:Teff ratio shown to elicit effective Treg-based suppression [65, 66]. What has to be taken in account is the amount of Teff that is expected within the recipient. Without lympho-depleting pre-treatment, it is stated that 49-79 × 10^9^ Tregs are needed to reach clinically efficacious numbers. If immunosuppressive drugs such as anti-thymocyte globulin (ATG) are administered however, it is suggested that 3-5 × 10^9^ Tregs are sufficient [66]. First routine *ex vivo* expansion cultures for polyclonal Treg products used beads coated with anti-CD3 and anti-CD28 in the presence of high-dose IL-2. Subsequent, cell culture condition optimization protocols adding rapamycin and TGF-β suggested further improvements in purity of *in vitro* expanded Treg cultures. With the supplementation of rapamycin, a mammalian target of rapamycin (mTOR) inhibitor, potential contamination of the Treg cell product with Teff is mitigated due to insensitivity of Tregs to mTOR blockade [67]. In expansion culture protocols containing rapamycin and TGF-β, Tregs showed higher suppressive capacity and stable FOXP3 expression due to increased TSDR demethylation [68]. These polyclonally expanded Tregs exhibit a wide range of TCR specificities with their main effects relying on bystander immunosuppression trough antigen-independent mechanisms [69].

Besides, there is growing evidence suggesting that antigen-specific Tregs are more efficient in regulating immunological responses compared to polyclonal expanded Tregs [70]. These statements are based on the superior homing capacity of antigen-specific Tregs, allowing localized and more potent regulation of inflammatory processes [71]. A variety of approaches for antigen-specific Treg expansion have been developed over the last years. After discovering that murine naïve CD4 + T cells have potential to develop into iTregs if stimulated with IL-2 and TGF-β *in vitro*, antigen-specific Teff as source for antigen-specific iTregs came into the center of attention [72]. Yet, these iTregs do not show a stable suppressive phenotype if re-introduced into inflammatory environment and they are likely to re-gain their pro-inflammatory characteristics *in vivo* [73]. Transgenic (over)expression of FOXP3 via lentivirus-based transduction in antigen-specific Tconv, however, lead to high levels of stable FOXP3 expression accompanied by suppressive capacities *in vitro* [74, 75] as well as *in vivo* [76, 77]. In addition, these iTregs maintained stability in inflammatory *in vitro* as well as *in vivo* conditions [77]. This approach requires retroviral transduction techniques which are associated with not only safety concerns, but also high production costs and vector capacity constraints [78]. However, one safety risk, the random insertion of FOXP3, was successfully addressed recently by utilizing advanced genetic tools such as TALEN or CRISPR/Cas9 to induce high expression of FOXP3 in Teff cells via homology directed repair (HDR) genome editing [79, 80].

Besides, different approaches of allospecific *in vitro* priming were developed. Putnam et al., for example, made use of allogeneic DCs whereas Sagoo et al. utilized donor-specific B cells to generate human Tregs with direct allospecificity *in vitro*. In both studies donor-specific Tregs succeeded over polyclonal Tregs in protecting human skin xenografts from alloimmune response-mediated injury [71, 81]. In addition, Jiang et al. made use of human leucocyte antigen A2 (HLA A2) peptide (138-170aa) pulsed immature DCs to generate human Tregs with defined antigen specificity via indirect recognition and demonstrated cell-contact-dependent effective suppression of Tconv cells [82]. Studies performed in murine skin allograft or GvHD models obtained similar results, strengthening the hypothesis of better efficacy of donor-specific Tregs [65, 83, 84]. Moreover, it was demonstrated that due to antigen-specificity, lower Treg cell numbers are needed to achieve sufficient suppression compared with polyclonal Tregs [71, 81].

Regardless of the fact that antigen-specific Tregs are more potent than polyclonally expanded Tregs, the main limitation of generating these cells is the low precursor frequency as well as challenging cell culture requirements. Another approach to generate targeted immunosuppressive Tregs is to introduce antigen-specific transgenic TCR or synthetic chimeric antigen receptors (CAR) into polyclonal Tregs. Utilizing genetically modified α and β TCR chains to obtain Tregs with a certain antigen-specificity already achieved promising results in autoimmune diseases [85, 86], GvHD [84], and transplantation [87]. With regard to the latter, it was demonstrated that engineered Tregs were even more efficient at tolerance induction when not only transduced with a TCR specific for direct but also indirect allorecognition in a murine heart allograft model (BALB/c →C57BL/6) [88]. These results again highlight the clinical potential of genetically engineered Tregs. However, there are some limitations to this approach such as the transduction of antigen-specific TCRs isolated from Teff into Treg.

### *In vivo* stimulation

Since *in vitro* expansion and adoptive Treg transfer require advanced GMP-compliant cell culture conditions, are accompanied by high costs and risk of contamination, different approaches for *in vivo* stimulation of Tregs are part of ongoing investigations.

#### Low dose IL-2

Treg survival as well as stability and function are dependent on (exogenous) IL-2 [89] and IL-2 deficiency results in Treg apoptosis [90]. Therefore, IL-2 became an interesting therapeutic option for enhancing Treg efficacy *in vivo*. In fact, low dose IL-2 therapy resulted in preferential expansion and activation of Tregs, whereas high dose treatment led to expansion of NK cells as well as cytotoxic T lymphocytes (CTL) and caused serious side effects [91, 92]. These observations are based on the expression of IL-2 receptors with different IL-2 binding affinities. Whereas CTLs only express β and γ receptor subunits resulting in low IL-2 binding affinity, Tregs express the IL-2 high-affinity receptor complex consisting of the α, β, and γ chains enabling activation and expansion even with low IL-2 availability [93]. Utilizing low-dose IL-2 administration, promising results have been demonstrated in clinical trials of patients with hepatitis C virus-induced vasculitis [94], GvHD [95], as well as Type 1 diabetes (T1D) [96]. In addition, Tahvildari et al. showed that low-dose IL-2 treatment led to increased levels of Tregs with only mild expansion of Teffs in a murine model of corneal transplantation and therefore improved allograft survival [97]. Clinical use of IL-2 therapy is however limited by the short serum half-life and dose-dependent toxicities [98, 99].

#### IL-2 complexes

Almost 30 years ago, Finkelman et al. could show that the complexation of cytokines and respective neutralizing anti-cytokine antibodies would increase the half-life and biological effect of cytokines *in vivo* [100]. A decade later, Boyman and Sprent discovered that IL-2 complexed to a specific anti-IL-2 monoclonal antibody, namely JES6-1, increased not only the duration and magnitude of the cytokine response but was able to selectively stimulate target cells. This approach predominantly led to the expansion of Tregs with only minor increase of (IL-2 responsive) CTLs and NK cells. This effect was suggested to be based on the binding of the JES6-1 antibody to a site of IL-2 that is essential for the interaction with the β (CD122) but not α (CD25) IL-2 receptor subunit, favoring the cytokine binding towards the IL-2 high affinity receptor, preventing potential side-effects seen in IL-2 high dose therapy [93]. The potential of IL-2/JES6-1 complexes to increase Treg levels and therefore decrease inflammation was already demonstrated in a murine model of fully mismatched islet cell transplantation. In fact, after 3 consecutive days of injecting IL-2/JES6-1 complexes, Treg levels within CD4 + T cells of the spleen were increased 5-fold and resulted in indefinite acceptance of the majority of grafts [101]. More importantly, it was demonstrated that administration of IL-2/JES6-1 complexes synergize with rapamycin and a short-term treatment of anti-IL-6 to significantly prolong survival of fully mismatched skin grafts (BALB/c →C57BL/6) [102]. Recently, researchers have been able to develop specific human anti-IL-2 receptor antibodies, demonstrating selective *in vivo* Treg expansion and suppressive potency in a mouse model of T1D, experimental autoimmune encephalomyelitis and xenogeneic GvHD [103]. These promising findings might pave the way for future IL-2 complex-based clinical trials [104].

### Polyclonal vs. antigen-specific Tregs

Whether polyclonal or antigen-specific Tregs are the future of Treg-based treatment remains another controversial topic regarding adoptive Treg transfer approaches. In fact, pre-clinical studies demonstrated that antigen-specific Tregs are more potent at inhibiting Teff *in vitro* as well as *in vivo* if compared to polyclonal Tregs [70]. However, studies on non-human primates suggesting lower efficacy of *ex vivo* induced antigen-specific Tregs with evidence of loss of regulatory mechanisms upon *in vivo* introduction [105]. In addition, antigen-specific Treg development requires challenging cell culture conditions as seen in the ARTEMIS trial where generation of the Treg product failed in 5 out of 10 patients enrolled in this study (NCT02474199). These results ignite the discussion whether polyclonal Tregs are more feasible for clinical translation of this therapeutic approach. Although it is stated that the bystander suppressive capacity of polyclonal expanded Tregs may cause increased susceptibility to opportunistic infections or tumor development due to unspecific suppression of immune responses and therefore limit the effect in transplantation settings [69, 106], human trials did not detect signs of over-immunosuppression after polyclonal Treg transfer. In addition, no events of Tregs converting into donor-specific Teff occurred in kidney transplant recipients, supporting the feasibility of polyclonal over antigen-specific Tregs [107, 108].

## Next generation approaches

### Engineered Tregs: CARs and TRUCKs

Another approach to confer antigen specificity is the expression of specific chimeric antigen receptors (CAR)s on immune cells. CARs are chimeric fusion proteins containing an extracellular antigen binding site of a monoclonal antibody fused to T-cell stimulatory and costimulatory intracellular domains [109]. One major advantage of CAR T cells compared with TCR transgenic T cells is the ability to recognize specific antigens without the requirement of antigen processing and presentation, independent of MHC classes I and II. Utilizing CAR-engineered Teff cells led to promising results in blood cancer settings [110, 111] which also gave rise to the development of advanced CAR constructs. The introduction of an intracellular costimulatory domain in addition to the single CD3ξ intracellular signaling domain, for example, resulted in better T-cell activation and proliferation [112]. More importantly, it has been demonstrated that Tregs engineered with this ‘second-generation’ CARs targeting HLA-A2 successfully prevented xenogeneic GvHD in a humanized mouse transplantation model *in vivo* [113]. In addition, precise antigen-specific action of HLA-A2 targeting CAR Tregs was verified in a side-to-side skin transplantation model demonstrating prolonged survival of HLA-A2 expressing grafts while no effect was seen for HLA-A2 non-expressing transplants [114]. In addition, migration of Tregs towards the desired site of impact has been shown to be crucial for therapeutic Treg-mediated suppression in transplantation [115]. It was demonstrated that donor-specific CAR Tregs are able to migrate into the targeted tissue where they are capable of not only delaying skin graft rejection but also decrease B cell responses and donor specific antibody production, whereas allospecific memory and graft rejection in sensitized mice was not attenuated [116]. Besides, Tregs are capable of exerting bystander suppression in inflammatory sites without direct targeting of cell surface antigens. This was demonstrated using CAR Tregs specific for citrullinated vimentin (CV), a protein abundantly and almost exclusively found in the inflamed joint extracellular matrix in rheumatoid arthritis (RA) patients. In detail, CV-specific CAR Tregs were able to proliferate when co-cultured with synovial fluid from the joints of RA patients, suggesting that CV within the extracellular matrix was sufficient to activate CAR Tregs specific for CV [117]. This approach offers the opportunity of using the bystander suppressive capacity of Tregs in inflammatory settings where direct targeting of antigen expressing cells might be disadvantageous based on reported cytotoxic activity of CAR Tregs in specific cases [43]. In addition, there are ongoing investigations regarding third- and fourth-generation (known as TRUCK) CAR Tregs that include further costimulatory domains or co-express cytokines and transcription factors in order to maximize the potential of CAR Tregs for individual disease treatment [118]. At present CAR Treg production is dependent mainly on γ-retroviral or lentiviral vectors known to be accompanied by not only safety concerns but also high manufacturing costs [78]. Nevertheless, first human clinical trials are now evaluating the safety and feasibility of HLA-A*02 recognizing CAR Tregs in HLA-A2 mismatched liver (NCT05234190) and kidney (NCT04817774) transplant recipients.

### Reducing off-target effects for *in vivo* Treg expansion: the orthogonal IL-2R/IL-2 system and the SynNotch receptor system

To further minimize the risk of side effects caused by immunosuppressive therapy or IL-2 administration for *in vivo* Treg expansion, Tregs expressing engineered IL-2 receptors that only engage with specific engineered IL-2 cytokines enable precise treatment approaches. It was already demonstrated that T cells transduced with an orthogonal IL-2Rβ subunit selectively bind a mutant IL-2 showing only minor interaction with wild-type IL-2Rβ *in vivo*. Furthermore, adoptive cell transfer (ACT) of orthogonal IL-2Rβ transduced effector CD4 + and CD8 + T cells resulted in elevated anti-tumor immune responses upon orthogonal IL-2 injection in a pre-clinical cancer model [119]. Recently, efficacy of orthogonal IL-2 receptor engineered Tregs was tested in a murine mixed chimerism model. It was demonstrated that administration of orthogonal IL-2 significantly increased orthogonal Treg numbers *in vivo* without promoting the expansion of other T-cell subsets. More importantly, this approach led to promising results involving donor hematopoietic cell engraftment with heart allograft acceptance at a later time point [120].

Another option to further improve Treg-based therapy could be the recently developed synthetic Notch (SynNotch) receptor system containing the Notch receptor regulatory core domain fused to custom-made extracellular recognition and intracellular transcriptional domains [121]. Upon synNotch receptor activation via target antigen binding the specific intracellular transcription factor is released and enters the nucleus in order to initiate expression of a certain gene. This system is completely independent of T-cell-based signaling pathways allowing customized antigen-specific T-cell response programs including the production of certain cytokines, therapeutic mediators, regulating transcription factors, or other immune response modulating adjuvants [122]. Promising results have already been obtained in studies on murine cancer models utilizing synNotch Teff cells [122] pathing the way for application in other immune cell types such as Tregs. Here, the synNotch system could be used in combination with the orthogonal IL-2R engineered Tregs to produce orthogonal IL-2 in an antigen-dependent positive feedback loop [117].

### Cytokine engineering: IL-2 muteins/IL-2 superkines

Engineering IL-2 to not only extend its half-life, but also improve its effectiveness *in vivo* led to the development of IL-2 ‘superkines’ or IL-2 muteins. The combination of screening IL-2 mutants for superior interaction with the β- and γ-subunit of the IL-2 receptor and developments of chimeric proteins by fusion of the cytokine to proteins like albumin or IgG for half-life extension, already led to promising results in cancer studies [104, 123, 124]. Recent development of IL-2 muteins with preferred binding to the α (CD25) IL-2 receptor subunit for favored interaction with the high-affinity IL-2 receptor represents another *in vivo* approach for selective Treg expansion. These new IL-2 muteins resemble the biological function of IL-2/JES6-1 mab complexes with promising results in treatment of type I diabetes in a pre-clinical murine model [125]. Moreover, there are ongoing clinical trials evaluating the safety and efficacy of Efavaleukin Alfa, a human IL-2 mutein Fc fusion protein selectively expanding human Tregs *in vivo*, being evaluated in GvHD (NCT03422627) and systemic lupus erythematosus (NCT03451422).

### Tackling the humoral response: BAR T cells

Recently, new chimeric immune receptor (CIR) T cells with specificities against antibody-producing B cells were developed. These B-cell antibody receptor (BAR) T cells consist of an antigen or antigen fragment which can be recognized by certain B cell receptors on the cell surface fused to an intracellular costimulatory or T-cell signaling domain [117]. Like CAR Tregs, BAR T cells recognize antigens independent of MHC, resulting in the suppression of antigen-specific B cells. This was already demonstrated in pre-clinical mouse models of allergy [118] as well as hemophilia A [119]. In transplantation settings, humoral alloimmune responses still account for the majority of late graft loss due to DSA development [120] and chronic humoral rejection. Thus, HLA-BAR T cells might be an attractive treatment approach as these cells could be utilized for the prevention of *de novo* DSA development through suppression of allospecific B cells [121]. In addition, effective recipient desensitization protocols for preformed DSA clearance remain wanted. Therefore, it was suggested that CD8 + T cells transduced with HLA-specific BAR in combination with drugs accounting for global plasma cell depletion could be a promising application for desensitization of patients with preformed DSA by targeting donor HLA-specific memory B cells [121]. What remains an uncertainty concerning the therapeutic use of BAR T cells is the fact that the alloantigen expressed on its cell surface might be recognized by recipient leucocyte T-cell receptors. This would result in destruction of BAR T cells and could potential trigger a ‘cytokine storm’ due to systemic inflammatory processes leading to excessive T-cell activation [121, 123]. In order to translate this approach into a clinical setting these limitations have to be addressed first.

### ‘Off the shelf’ Tregs

High costs accompanied to GMP-compliant *in vitro* Treg expansion together with low baseline autologous cell numbers still are one of the limiting factors for large scale application of adoptive Treg therapy. This problem is even more pronounced in transplant patients receiving immunosuppressive treatment for underlying autoimmune diseases or chronical illness as well as patients on the waiting list for re-transplantation. As a result, the development of ‘off-the shelf’ T-cell products remains in the center of scientific attention. Different approaches have been investigated over the years including third party Tregs obtained from UCB or donor bone marrow-derived endogenous Tregs [126] with the goal of creating a biobank for Tregs for maximum MHC matching. Having a Treg biobank with ready-to-use Tregs at any time would expand the availability of Treg therapies from living-donation settings to recipients of DBD and DCD donors. After transfer however, extrinsic Tregs might still face the problem of host-mediated elimination due to the expression of foreign MHC molecules. The approach of using the CRISPR-Cas9 system to achieve HLA deficient Tregs led to NK cell-mediated elimination of these cells due to lack of canonical HLA molecules on the cell surface. The expression of non-canonical HLA-E or HLA-G, which are NK inhibitory receptor ligands, could be one possibility of solving this issue [127]. Besides, the expression of CD47, commonly known as the ‘do-not-eat-me-signal’ for macrophages, was suggested to be of additional benefit in this approach [128].

‘Off the shelf’ CAR Tregs could represent another opportunity to circumventing the need for high quantity autologous Tregs and challenging cell culture conditions to generate antigen-specific Tregs. CAR Tregs can be generated fast and in large numbers from naive or polyclonal expanded Tregs and are, as mentioned above, able to recognize antigens presented within the MHC and in a non-MHC restricted manner. After *in vivo* transfer, however, off-target effects due to missing the intended destination might be an issue. Thus, including suicide genes acting as ‘safety switch’ in case of pan-immunosuppression could be a solution. RQR8 or huEGFRt have been successfully tested as suicide genes in murine models where cells expressing these surface proteins were targeted and sufficiently deleted by the respective monoclonal antibody rituximab/cetuximab [129, 130] and are now part of a CAR-Treg human liver transplantation trial (NCT05234190). For prevalent clinical application of CAR Tregs, however, some other safety concerns remain. Even though to date no issues regarding viral-based transfer of the CAR to Tregs were reported, there is still the need for alternative gene-editing tools such as CRISPR-Cas9 or TALENs since testing and production of viral vectors is not only time consuming but also expensive. Moreover, random genome insertion of the transgene is a negative property of viral vectors that can cause oncogenic genetic changes. However, utilizing CRISPR-Cas9 or TALENs enables the replacement of endogenous TCR with a specific CAR if targeting the endogenous T-cell receptor-α constant (TRAC). These modifications would minimize the risk of side-effects such as alloreactivity resulting in GvHD, facilitating the development of ‘off the shelf’ CAR T-cell products [131, 132]. Another approach to reach this aim is the use of modular or universal CAR. Pierini et al. for example developed a murine CAR consisting of an anti-FITC scFv portion fused to a CD28 and CD3 co-stimulatory domain, termed mAbCAR. This synthetic receptor is suggested to bind any FITC conjugated mAb enabling fast generation of CAR Tregs directed against a variety of antigens. The efficacy of this system was demonstrated in a murine model by significantly prolonging the survival of fully mismatched islets and skin grafts if H-2Dd-mAbCAR Tregs were injected [133]. These promising results display a first step towards another valuable tool for antigen-specific ‘off the shelf’ Treg therapy.

## Clinical experience

Many clinical trials have already introduced Treg cellular therapy into clinical trials to treat autoimmune diseases, hematopoietic stem cell transplantation and SOT recipients [134, 135]. The clinical experience with Treg therapy in SOT in ongoing and already published clinical trials is summarized in Table 1.

**Table 1:** ongoing clinical trials adopting regulatory T-cell therapy in solid organ transplantation (search date September 15, 2022)

Study ID	Phase	Age	Title	Product	Dose	Status	Location
Renal transplantation—endogenous Treg expansion							
NCT02417870	I/II	18-75 (adult, older adult)	Ultra-low Dose Subcutaneous IL-2 in Renal Transplantation	Low-dose recombinant IL-2 (proleukin)	—	Terminated (June 2021)	Brigham and Women`s Hospital, Boston, US
Renal transplantation—adoptive Treg therapy							
NCT02088931	I	18-50 (adult)	Treg Adoptive Therapy for Subclinical Inflammation in Kidney Transplantation (TASK)	CD4 + CD127lo/-CD25 + polyclonally expanded Tregs	3.2 × 10^8^	Completed (July 2022)	University of California, San Francisco, US
NCT02091232	I	>18 (adult, older adult)	Infusion of T-Regulatory Cells in Kidney Transplant Recipients (The ONE Study)	Tregs (recipient) stimulated with donor PBMCs and belatacept	4-9 × 10^8^	Completed (Nov 2021)	Massachusetts General Hospital, Boston, US
NCT04817774	I/II	18-70 (adult, older adult)	Safety and Tolerability Study of Chimeric Antigen Receptor T-Reg Cell Therapy in Living Donor Renal Transplant Recipients	CD4 + CD45RA + CD25 + CD127low/- HLA-A*02 specific CAR Tregs	—	Recruiting (Dec 2021)	University Hospitals Leuven Leuven, Belgium (and 3 other centers)
NCT03943238	I	18-65 (adult, older adult)	TLI, TBI, ATG & Hematopoietic Stem Cell Transplantation and Recipient T Regs Therapy in Living Donor Kidney Transplantation	Autologous polyclonally expanded Tregs	starting at 25 × 10^6^/kg	Recruiting (May 2022)	Stanford University Palo Alto, Northwestern University Chicago, US
NCT03284242	n/a	18-65 (adult, older adult)	A Pilot Study Using Autologous Regulatory T Cell Infusion Zortress (Everolimus) in Renal Transplant Recipients	Autologous polyclonally expanded Tregs	n/a	Recruiting (May 2022)	University of Kentucky Medical Center Lexington, Kentucky, US
NCT02711826	I/II	>18 (adult, older adult)	Treg Therapy in Subclinical Inflammation in Kidney Transplantation	Autologous polyclonally expanded Tregs	5.5 ± 4.5 × 10^8^	Recruiting (March 2022)	University of California at San Francisco, US (and 5 other centers)
NCT02145325	I	18-65 (adult, older adult)	Trial of Adoptive Immunotherapy With TRACT to Prevent Rejection in Living Donor Kidney Transplant Recipients	Autologous polyclonal expanded CD4 + CD25 + nTregs	0.5-5 × 10^6^	Completed (Oct 2019)	Northwestern University Comprehensive Transplant Center Chicago, Illinois, US
NCT03867617	I/II	>18 (adult, older adult)	Cell Therapy for Immunomodulation in Kidney Transplantation	Autologous polyclonally expanded CD4 + CD127lo/- CD25 + CD45RA Tregs	0.3-1.5 × 10^7^	Recruiting (Sep 2019)	Medical University of Vienna, Vienna, Austria
NCT01446484	I/II	1-18 (child)	Treatment of Children With Kidney Transplants by Injection of CD4 + CD25 + FoxP3 + T Cells to Prevent Organ Rejection	Autologous CD4 + CD25 + CD127low FoxP3 + Tregs	2 × 10^8^	Unknown (Nov 2011)	Russian state Medical University, Moscow, Russian Federation
NCT02371434	I/II	18-65 (adult, older adult)	The ONE Study nTreg Trial (ONEnTreg13)	Autologous polyclonally expanded CD4 + CD25 + FoxP3 + nTregs	0.5-3 × 10^6^	Completed (Feb 2020)	Charité University Medicine, Berlin, Germany
NCT02244801	I	18-70 (adult, older adult)	Donor-Alloantigen-Reactive Regulatory T Cell (darTreg) Therapy in Renal Transplantation (The ONE Study)	Donor alloantigen reactive Tregs (darTregs)	3 × 10^8^; 9 × 10^8^	Completed (Oct 2018)	University of California San Francisco, US
NCT02129881	I/II	>18 (adult, older adult)	The ONE Study UK Treg Trial	Autologous polyclonally expanded Tregs	1-10 × 10^6^/kg	Completed (Jan 2019)	Guy’s Hospital London, UK
ISRCTN 11038572	II	>18 (adult, older adult)	TWO study: cell therapy trial in renal transplantation	Autologous polyclonally expanded Tregs	5-10 × 10^6^/kg	Recruiting (June 2022)	Oxford Transplant Centre, Churchill Hospital, Oxford, UK
Liver transplantation—endogenous Treg expansion							
NCT02739412	II	18-65 (adult, older adult)	Efficacy of Low Dose, SubQ Interleukin-2 (IL-2) to Expand Endogenous Regulatory T-Cells in Liver Transplant Recipients	Low-dose recombinant IL-2 (proleukin)	0.30 MIU per meter squared body surface area; for 4 weeks	Active, not recruiting (May 2021)	Beth Israel Deaconess Medical Center Boston, Massachusetts, US
NCT02949492	IV	18-50 (adult)	Low-dose IL-2 for Treg Expansion and Tolerance (LITE)	Low-dose recombinant IL-2 (proleukin)	—	Terminated (Aug 2019)	Kings Collage Hospital London, UK
Liver transplantation—adoptive cell therapy							
NCT01624077	I	10-60 (child, adult)	Safety Study of Using Regulatory T Cells Induce Liver Transplantation Tolerance	Autologous polyclonally TGF-β induced CD4 + CD25 + CD127- Tregs	1 × 10^6^/kg	Unknown (Feb 2015)	Nanjing Medical University Nanjing, Jiangsu, China
NCT03654040	I/II	18-70 (adult, older adult)	Liver Transplantation With Tregs at UCSF	Autologous expanded donor alloantigen reactive Tregs (arTregs)	30-90 × 10^6^ total Treg cells	Recruiting (Aug 2021)	University of California, San Francisco San Francisco, California, US
NCT03577431	I/II	18-70 (adult, older adult)	Liver Transplantation With Tregs at MGH	Autologous expanded donor alloantigen reactive CD4 + CD25 + CD127- Treg cells (arTregs)	2.5-125 × 10^6^	Recruiting (Nov 2021)	Massachusetts General Hospital: Transplantation Boston, Massachusetts, United States
NCT02474199	I/II	18-70 (adult, older adult)	Donor Alloantigen Reactive Tregs (darTregs) for Calcineurin Inhibitor (CNI) Reduction (ARTEMIS)	Autologous donor alloantigen reactive Tregs (darTregs)	3-5 × 10^8^	Completed (Feb 2021)	University of California at San Francisco San Francisco, US Northwestern University Comprehensive Transplant Ctr Chicago, US Mayo Clinic in Rochester Rochester, US
NCT02188719	I	21-70 (adult, older adult)	Donor-Alloantigen-Reactive Regulatory T Cell (darTregs) in Liver Transplantation (deLTa)	Autologous donor alloantigen reactive Tregs (darTregs)	2.5-96 × 10^7^	Terminated (Sep 2020) - has results	University of California at San Francisco San Francisco, US Northwestern University Comprehensive Transplant Ctr Chicago, US Mayo Clinic in Rochester Rochester, US
NCT02166177	I/II	18-70 (adult, older adult)	Safety and Efficacy Study of Regulatory T Cell Therapy in Liver Transplant Patients (ThRIL)	Autologous polyclonally expanded Tregs	0.5-1; 3-4.5 × 10^6^/kg	Completed (Jan 2019)	Kings College Hospital London, UK
UMIN-000015789	I/II	18-65 (adult, older adult)	Tolerance induction by a regulartory T cell-based cell therapy in living donor liver transplantation	Donor-reactive Treg-enriched cell product	0.23-6.37 × 10^6^ Tregs/kg	Recruiting (until July 2012) Data published 2016 [136]	Hokkaidou University Graduate School of Medicine, Japan
NCT05234190	I/II	18-70 (adult, older adult)	Safety and Clinical Activity of QEL-001 in A2-mismatch Liver Transplant Patients (LIBERATE)	Autologous CAR Tregs targeting HLA-A2 (HLA-A2 CAR-Treg)	—	Recruiting (Feb 2022)	Cambridge University Hospitals NHS Foundation Trust Cambridge, UK Royal Free London NHS Foundation Trust London, UK King’s College Hospital NHS Foundation Trust London, UK

### Translational hurdles

Although the importance of Tregs for long-term survival of allografts has been demonstrated in several pre-clinical as well as clinical trials, there is still no single (pre-clinical) study showing that Tregs alone are sufficient to induce tolerance in immunocompetent hosts or even significantly prolong SOT survival. There are multiple clinical trials demonstrating the safety of adoptively transferred Tregs in combination with immunosuppressants in kidney as well as liver transplantation (Table 1). However, Treg therapy for the induction of a pro-tolerogenic state with intention to wean the patients off immunosuppressive drugs was successfully tested in only one clinical liver transplantation trial by Todo et al [136, 137]. This approach is pursued in further ongoing liver transplantation studies (NCT03654040; NCT03577431). The use of Treg therapy based weaning protocols in heart and lung transplantation, however, remains challenging due to the lack of reliable biomarkers or functional assays identifying tolerant patients and no applicable rescue options in case of treatment-based rejection [138]. Although several studies correlate increased Treg numbers with improved lung transplant function and protective effect against chronic lung allograft dysfunction in animals and humans, Treg therapy still requires in-advance preparation time [139, 140]. Since planned living organ donation is only feasible for kidney and liver transplantation, these treatment options remain challenging for heart and lung transplantation. Recently, it was shown in a rat model of lung transplantation that *in vitro* expanded recipient-derived Tregs administered to the transplant during *ex vivo* perfusion resulted in successful inhibition of early alloimmune responses post-transplantation, claiming to provide an approach suitable for clinical translation to lung transplantation from deceased donors [141]. However, whether Treg-mediated tolerance induction is sufficiently stable to endure integrity during other opportunistic infections throughout the patient’s life is pending. In addition, several studies suggest differences in success of graft acceptance depending on the type of organ transplanted, implying the need for organ-specific approaches. Whereas liver transplant patients (especially pediatric patients) are the most common to develop ‘operational tolerance’, late after transplantation, this is a really rare event in kidney transplantation. Skin and intestines are considered to be the most immunogenic organs [136, 142]; however, the detailed mechanisms responsible for this phenomenon remain controversial [143].

### Immunosuppressive drugs

As mentioned above, using autologous *ex vivo* expanded Tregs is time-consuming and does not allow fast application. Thus, it only can be implemented in planned living-donor transplant settings making this approach suitable for living-donor kidney and liver transplantation only. In addition, there are several studies demonstrating negative effects of transplantation-related immunosuppressive drugs on Treg cell numbers or Treg-related suppressive capacities, directly interfering with the feasibility of autologous Treg therapy. Calcineurin inhibitors, such as Tacrolimus or cyclosporine A, lead to decreased Treg numbers in liver and kidney transplant patients since this medication impedes IL-2 transcription which is crucial for survival and maintenance of Tregs. In a murine model of skin transplantation, it was shown that this treatment further results in less efficient Treg-mediated suppression of Teff [144]. Besides, there is evidence that co-stimulation blockade with CTLA4-Ig leads to decreased skin graft acceptance in a pre-clinical mouse model due to inhibition of IL-2 complex-mediated *in vivo* Treg expansion. Moreover a loss of suppressive function is suggested, based on limitation of crucial CD28-dependent signals [145]. On the other hand, *in vitro* studies on murine polyclonal-induced iTregs revealed no negative impact but rather improvement of Treg generation and suppressive function in the presence of the co-stimulation blocker CTLA4Ig [146]. Besides, there are studies stating that moderate CD80/CD86 blockade using CTLA4-Ig may be beneficial for pTreg development in both, rodents and humans [147, 148]. These suggestions are based on observations claiming that activation of Teff cells requires higher CD80/CD86 expression than Treg activation since partial blockade prevented Teff cells emergence, but maintained Treg homeostasis [147, 148]. Despite these controversial results, FDA approved costimulation blockers are used in patients with autoimmune disease and after renal transplantation. Further in-depth studies on the combination of Treg-based therapy with CTLA4-Ig are clearly needed.

Other immunomodulatory drugs commonly used in transplanted patients are suggested to favor *in vivo* Treg action. Methylprednisolone, for example, is a corticosteroid widely used to treat transplant patients facing acute rejection. The administration of this substance during kidney rejection also leads to an altered T-cell composition that is beneficial for a highly-suppressive DR^high^ CD45RA^−^ Treg population [149]. Furthermore, as anti-thymocyte globulin (ATG) gained interest as induction therapy in transplantation setting in the last years, studies involving Tregs revealed that under low-dose ATG regimen, circulating *in vivo* Treg numbers in renal transplant recipients are reduced, but to a lesser extent than Teff, shifting the Treg:Teff ratio to a pro-tolerogenic setting. In addition, it was stated that recovery of Tregs was faster after ATG administration if compared to Teff [150]. Moreover, the FDA approved mTOR inhibitor rapamycin (sirolimus and everolimus) leads to inhibition of Teff cells while sparing Tregs *in vivo* as well as *in vitro*. In fact, a 4-fold higher amount of peripheral Treg has been detected in kidney transplant patients receiving sirolimus if compared to patients receiving cyclosporine [151]. However, it is noteworthy that this increase in Tregs is rather due to higher treatment sensitivity of Tconv and does not promote Treg expansion [152]. Nevertheless, combining rapamycin with Treg expansion therapy is a promising treatment approach.

## Concluding remarks

Pre-clinical studies reaching from *in vivo* and *ex vivo* expanded polyclonal to *in vitro* induced antigen-specific and genetically modified Tregs provided promising results for the improvement of long-term graft survival in context with immunosuppression minimization. Multiple approaches of Treg-based therapy that are now being evaluated for efficacy and safety in human trials. Whereas the ONE study consortium and others demonstrated feasibility and safety of multiple regulatory cell products [56, 153], to date there is only one published study to prove efficacy of Treg therapy in clinical transplantation [136].

The enhancement of Treg function by introducing CARs with tissue specific chimeric receptors, a FOXP3 phenotype lock module as well as a ‘safety switch’ is now part of a human phase I/II liver transplantation clinical trial (NCT05234190).

However, there are still plenty of open questions regarding Treg stability, Treg source, specificity, mode of action and migration toward the intended site of action. Approaches aiming for ‘off the shelf’ universal Tregs or ‘delayed tolerance’ approaches would furthermore expand their therapeutic potential for organ donation after circulatory death (DCD) and brain death (DBD) settings. Recent developments and new strategies such as genetic engineering tools will further enhance the potential of Treg cells as immunotherapy to minimize/avoid conventional immunosuppression and increase quality of life of transplant patients.

## Data Availability

Not applicable.

## Abbreviations

ACT: adoptive cell transfer
APC: antigen-presenting cell
ATG: anti-thymocyte globulin
BAR: B-cell antibody receptor
CAR: chimeric antigen receptor
CIR: chimeric immune receptor
CTL: cytotoxic T lymphocyte
CTLA-4: cytotoxic T lymphocyte antigen 4
DBD: donation after brainstem death
DCD: Organ donation after circulatory death
DSA: donor-specific antibody
FACS: fluorescence activated cell sorting
GMP-compatible: good manufacturing practice compatible
GvHD: graft versus host disease
HDR: homology directed repair
HLA: human leucocyte antigen
IPEX: immunodysregulation polyendocrinopaty enteropathy X-linked syndrome
MHC: major histocompatibility complex
mTOR: mammalian target of rapamycin
NK: natural killer cell
NRP1: neuropilin 1
PBMCs: peripheral blood mononuclear cells
SOT: solid organ transplantation
TCR: T-cell receptor
Teff: effector T cells
TRAC: T-cell receptor-α constant
Treg: regulatory T cell
TSDR: Treg-specific demethylation region
UCB: umbilical cord blood

## Permission to Reproduce

Manuscript does not use any third party copyrighted material.
